# Facet joint fusion in lumbar surgery: a biomechanical review of posterior column preservation and reconstruction

**DOI:** 10.3389/fsurg.2026.1918664

**Published:** 2026-10-06

**Authors:** Tong Xu, Xingyu Zhao, Lei Zhang, Kai Wang, Zhenlei Liu, Shaocheng Liu, He Wang, Yaobin Wang, Yuhua Yang, Weikang Zhang, Hao Wu

**Affiliations:** 1 Department of Neurosurgery, Xuanwu Hospital, Capital Medical University, Beijing, China; 2 Department of Critical Care Medicine, Beijing Mentougou District Hospital, Beijing, China

**Keywords:** adjacent segment disease, interbody fusion, lumbar facet arthrodesis, pedicle screw fixation, posterior column injury, spinal biomechanics, two-column fixation

## Abstract

Adjacent segment disease (ASD) is a multifactorial long-term complication of lumbar interbody fusion. In addition to patient-related factors, pre-existing degeneration, sagittal alignment, and fusion-related changes in construct stiffness, iatrogenic disruption of the posterior elements may contribute to altered adjacent-segment biomechanics. The role of the facet joint complex in this process has received comparatively limited attention. This narrative review synthesizes biomechanical, cadaveric, and clinical evidence concerning lumbar facet arthrodesis, posterior-column preservation and reconstruction, and two-column fixation after lumbar interbody fusion. Biomechanical studies suggest that preservation of the posterior ligamentous and facet structures can influence segmental stability, load sharing, and adjacent-level intradiscal or facet stresses. Facet-based fixation provides rotational constraint under experimental conditions and may offer a different stiffness profile from conventional pedicle screw constructs. The potential role of facet arthrodesis appears to depend on the extent of posterior-element disruption, residual instability, and the stability requirements of the overall construct. Current evidence is insufficient to recommend its routine use after TLIF or PLIF or to conclude that it reduces symptomatic ASD, reoperation rates, or improves long-term patient-reported outcomes. Prospective clinical studies with long-term follow-up are required.

## 1 Introduction

Adjacent segment disease (ASD) is a well-recognized long-term complication after lumbar interbody fusion, characterized by new or progressive pathology at motion segments adjacent to the fused level (1). Fusion alters regional load sharing and motion patterns, which may contribute to degenerative changes at adjacent segments. Radiographic adjacent-segment degeneration can include disc degeneration, facet arthropathy, instability, or spondylolisthesis, whereas clinically significant ASD refers to symptomatic pathology that may present with axial low back pain, radiculopathy, or neurological deterioration and, in some patients, require revision surgery (2).

The clinical burden of ASD is substantial. In a cohort of 5,291 patients undergoing lumbar interbody fusion, the overall reoperation rate for symptomatic ASD was 8.37% at a mean follow-up of 6.3 years (3). In selected series, early reoperation rates within two years have been reported at 8.0%–9.1% (4). Radiographic adjacent-segment degeneration is considerably more common, with reported rates ranging from 27.8% to 84%, depending on follow-up duration and diagnostic criteria (5–7). Recent advances in cage design and minimally invasive surgical techniques have improved endplate coverage and restoration of segmental alignment. Although some studies suggest that these approaches may reduce radiographic adjacent-segment degeneration (8, 9), whether they reduce symptomatic ASD or long-term revision rates remains uncertain (10).

Most previous work has focused on anterior cage design, sagittal alignment, and minimally invasive access, whereas the contribution of posterior-element integrity has received comparatively less attention. The intervertebral disc and paired facet joints form a three-joint complex that contributes to load transmission, motion guidance, and segmental stability (11). Surgical disruption of this complex through facetectomy, capsular injury, or ligamentous resection can alter the mechanical behavior of the motion segment. Finite element studies have shown that facetectomy increases segmental motion, intradiscal pressure, and facet loading under selected loading conditions (12), while fusion may further alter loading at adjacent facet joints (13). Mechanical loading has also been associated with biological responses within the ligamentum flavum, including pathways involved in tissue hypertrophy and degeneration (14). More recent finite element studies suggest that disruption of the posterior ligamentous complex may further increase adjacent-level annular stress and intradiscal pressure beyond the effect of fusion alone (15, 16). These findings support the concept that posterior-element disruption may represent one potentially modifiable surgical contributor to postoperative biomechanical changes, although its independent contribution to clinical ASD remains uncertain.

Against this biomechanical background, facet-based posterior fixation using trans-facet screws or dedicated facet implants has been proposed as a means of supplementing interbody support and potentially restoring part of the posterior stabilizing function. However, several questions remain unresolved. First, it is unclear whether facet arthrodesis can provide sufficient stability as an alternative to pedicle screw fixation in selected settings (17) or should primarily be regarded as an adjunct. Second, different lumbar fusion approaches produce different patterns and degrees of posterior-element disruption (18), but no validated classification currently links these injury patterns to specific fixation strategies. Finally, much of the supporting evidence derives from finite element and cadaveric studies (19, 20), and the extent to which these biomechanical findings translate into higher fusion rates, improved patient-reported outcomes, lower rates of symptomatic ASD, or fewer reoperations remains uncertain.

This review examines the relationship between lumbar fusion approaches, posterior-element preservation or disruption, and the potential role of facet arthrodesis. Specifically, we aim to: (1) clarify the complementary rather than interchangeable biomechanical roles of facet-based and pedicle screw fixation; (2) propose a conceptual framework for considering supplementary facet-based fixation according to the pattern and extent of posterior element preservation or disruption; and (3) distinguish biomechanical plausibility from established clinical benefit while identifying the principal limitations of the current evidence and priorities for future research.

## 2 Method

This narrative review was conducted in accordance with published clinical and biomechanical evidence; formal ethical approval was not required.

### 2.1 Inclusion and exclusion criteria

Given the methodological heterogeneity among finite element studies, cadaveric investigations, and clinical trials, evidence integration adhered to the principle of qualitative hierarchical stratification: conclusions derived from randomised controlled trials (RCTs) were assigned the highest evidentiary weight, followed by large-sample prospective cohort studies, cadaveric biomechanical experiments, and finite element simulations. Where conflicts existed between finite element conclusions and clinical data, precedence was accorded to the latter and the discrepancy explicitly annotated.

Inclusion criteria encompassed: (1) studies investigating the anatomy and biomechanical characteristics of the lumbar facet joints; (2) studies comparing biomechanical or clinical outcomes across different lumbar fusion procedures; (3) studies evaluating dedicated facet arthrodesis devices or trans-facet screw fixation systems; and (4) study designs spanning finite element analysis, cadaveric experiments, and clinical investigations. Exclusion criteria comprised: (1) non-lumbar spinal studies; (2) studies lacking quantitative biomechanical or validated clinical outcome data; and (3) conference abstracts, editorial commentaries, or opinion-based publications.

### 2.2 Information sources

Following establishment of the eligibility criteria, a comprehensive systematic search was independently conducted across multiple electronic databases, including PubMed, CNKI, and Wanfang Database. Articles were screened sequentially by title, abstract, and full text. Reference lists of all included studies were manually searched to identify potentially eligible publications not captured by the primary electronic search.

### 2.3 Search strategy

The search strategy was developed in accordance with PubMed MeSH guidelines. To minimise the risk of omitting relevant literature, controlled vocabulary (MeSH terms) was combined with free-text keywords encompassing ‘facet joint’, ‘lumbar fusion’, ‘adjacent segment degeneration’, ‘posterior column’, ‘biomechanics’, ‘facet arthrodesis’, ‘pedicle screw’, and ‘two-column fixation’. Boolean operators (AND, OR) were used systematically to construct search strings, and strategies were adapted to the syntax requirements of each database.

### 2.4 Selection process

Study selection was conducted independently by two authors in strict accordance with the predefined eligibility criteria, proceeding through sequential stages of title/abstract screening followed by full-text appraisal. Disagreements regarding eligibility were resolved through structured discussion with a third reviewer until consensus was reached.

## 3 Results

### 3.1 Study selection

The comprehensive database search retrieved a total of 1,927 articles. Following sequential screening by title, abstract, and full text in accordance with the predefined inclusion and exclusion criteria, 57 articles met eligibility requirements and were included in the final synthesis (54 in English and 3 in Chinese).

### 3.2 Anatomy and functional role of the lumbar facet joints

The lumbar facet (zygapophyseal) joints are paired synovial joints located posterolaterally within each motion segment, formed by the inferior articular processes of the cranial vertebra and the superior articular processes of the caudal vertebra (21, 22). Their three-dimensional orientation varies by spinal level and between individuals, influencing motion guidance and resistance to rotation and shear (11, 22–24). Facet orientation and tropism have also been associated with patterns of lumbar degeneration (25, 26). Together with the intervertebral disc, the paired facet joints form the three-joint complex, in which the anterior and posterior structures jointly contribute to load transmission and segmental stability (Figure 1).

**Figure 1:**
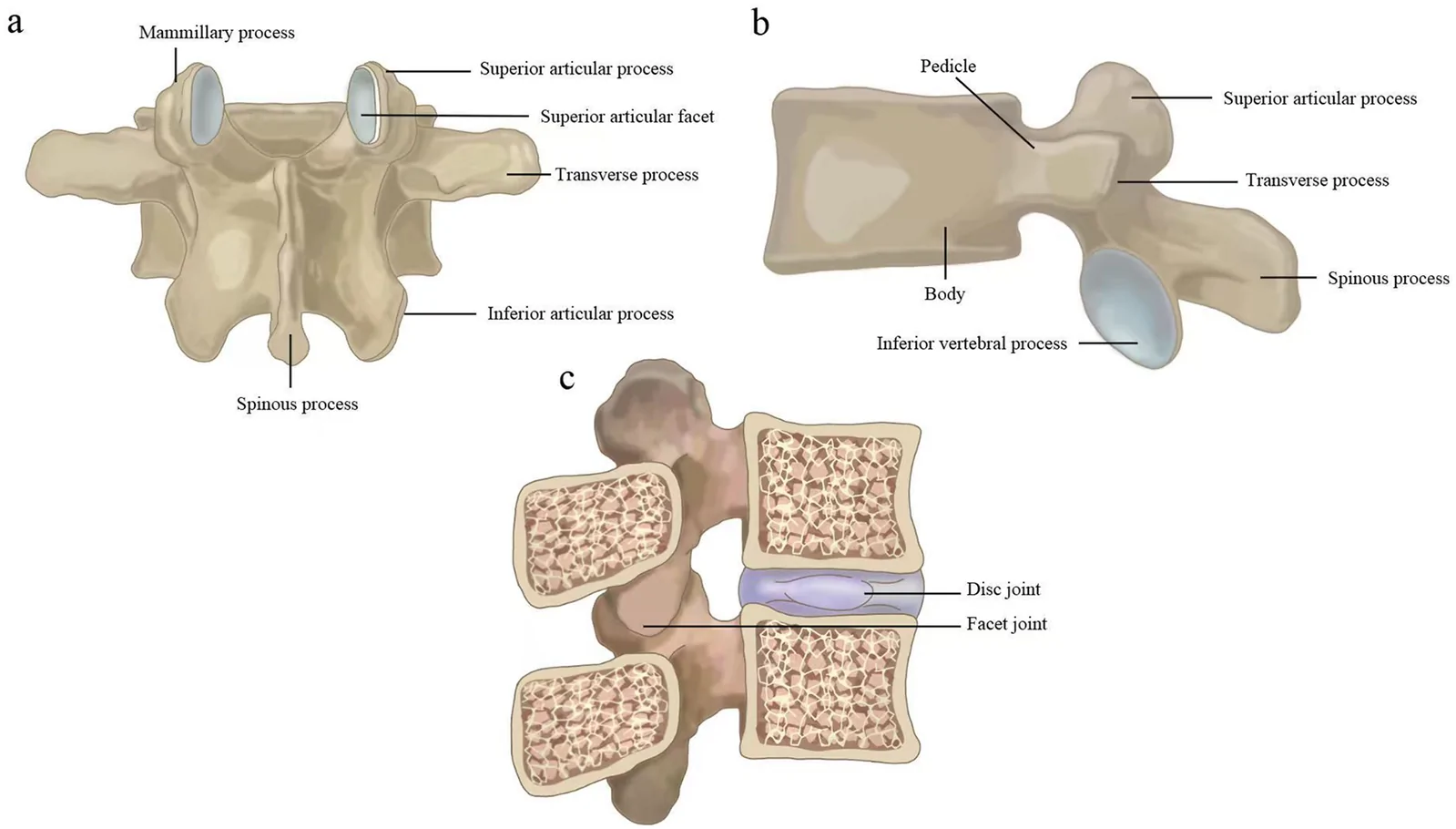
Anatomy of the lumbar spinal motion segment and three–joint complex. **(a)** Posterior osseous anatomy of a lumbar vertebra demonstrating the superior articular process, transverse process, pedicle, and spinous process. **(b)** Lateral view illustrating the inferior articular process, intervertebral disc, and facet joint. **(c)** Parasagittal view showing the intervertebral disc and paired facet joints forming the three–joint complex.

The mechanical contribution of the facet joints varies with posture, loading direction, and disc condition. Yang and King reported that normal lumbar facets carried approximately 3%–25% of axial compressive load under experimental conditions, with loads as high as 47% observed in arthritic facets (27). Adams and Hutton found that approximately 16% of intervertebral compressive load was transmitted through the facet joints in simulated erect standing, whereas facet loading was substantially reduced in flexed sitting (28). These findings illustrate that facet load sharing is condition dependent rather than fixed.

In addition to axial load sharing, the facet joints contribute to restraint of excessive rotation and translation. Their contribution becomes particularly relevant during extension, axial rotation, and combined loading, when articular contact forces may increase (11, 22, 29). The facet joints should therefore be regarded as one component of the integrated stabilizing system of the lumbar motion segment rather than as the sole or dominant determinant of stability. Surgical facetectomy or capsular injury can alter this mechanical contribution, but the magnitude of the effect depends on the extent of resection and the condition of the remaining disc, ligamentous structures, and instrumentation.

### 3.3 Posterior element disruption and load redistribution after lumbar fusion

Lumbar fusion alters motion and load sharing at both the treated and adjacent levels. Radiographic adjacent segment degeneration has been reported in 36%–84% of patients during long-term follow-up, although its occurrence is influenced by multiple patient-, alignment-, and surgery-related factors (5). The extent to which posterior structures are preserved may further modify these postoperative biomechanical changes.

The posterior ligamentous structures, including the supraspinous and interspinous ligaments and ligamentum flavum, contribute to passive restraint of the lumbar motion segment (30, 31). Disruption of these structures, together with partial or complete facetectomy, can alter segmental motion and load transmission. At the same time, fusion and instrumentation increase stiffness at the treated level and may redistribute motion and loading toward adjacent segments (32). Finite element studies suggest that these two effects may interact. In one model, posterior ligamentous complex disruption increased adjacent annular stress by more than 30% and intradiscal pressure by more than 40% during flexion (15). Cai et al. similarly reported greater adjacent level biomechanical changes when increased construct stiffness was combined with posterior structure damage than when either factor was modeled alone (16).

These findings support considering the condition of the posterior elements when interpreting postoperative biomechanics. However, changes in intradiscal pressure, annular stress, or segmental motion are biomechanical surrogate outcomes and do not establish that posterior structure disruption independently causes clinical ASD or that posterior column reconstruction prevents it. The following sections therefore compare commonly used lumbar interbody fusion approaches according to their typical patterns of posterior element preservation or disruption, while recognizing that the actual extent of injury varies with the surgical exposure, degree of decompression, facetectomy, and ligamentous resection (Figure 2).

**Figure 2:**
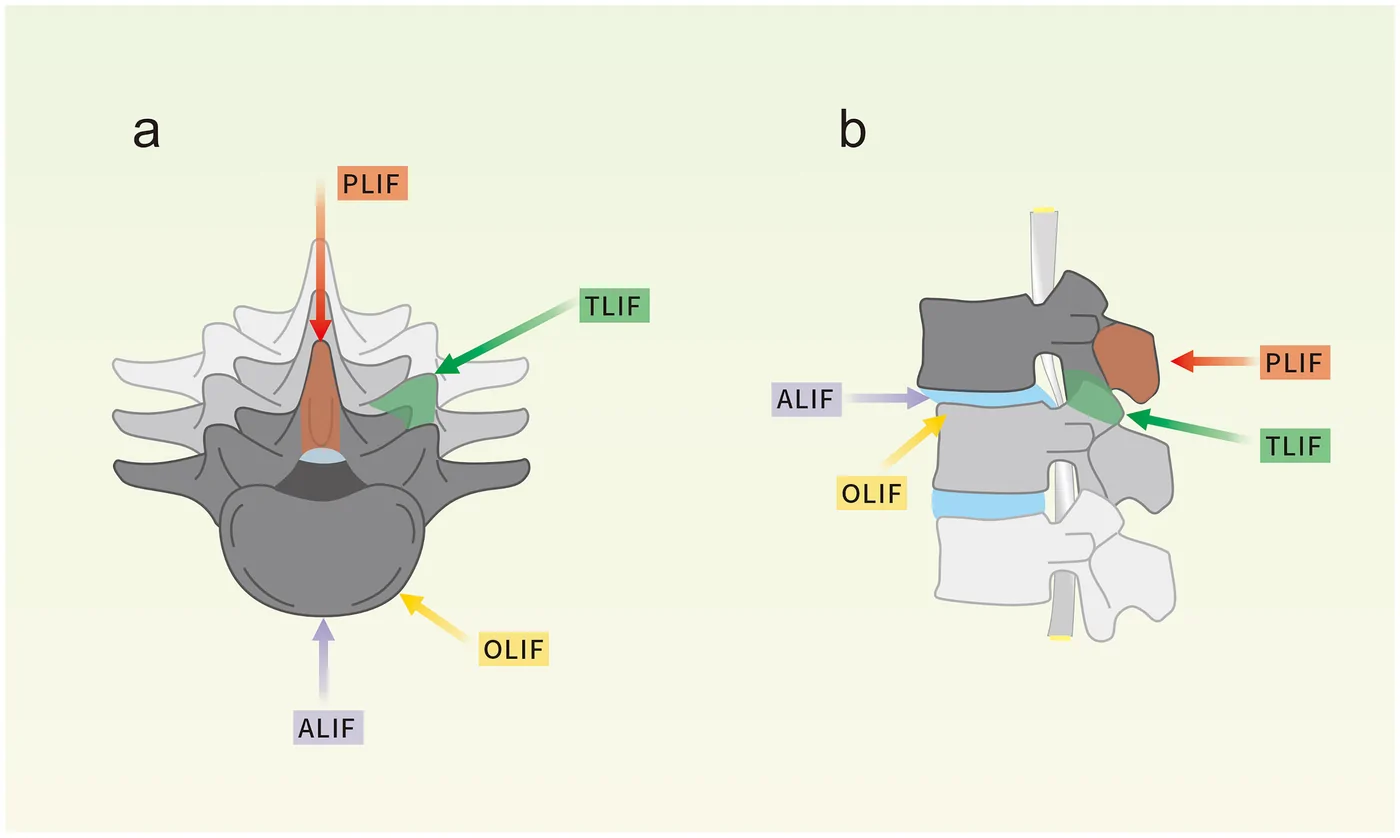
Axial and sagittal illustrations of common lumbar interbody fusion approaches and their typical patterns of posterior element preservation or disruption. **(a)** Axial comparison of ALIF, OLIF, TLIF, and PLIF. **(b)** Sagittal illustration of the corresponding surgical corridors and posterior element involvement.

### 3.4 Posterior element preservation in ALIF and OLIF

Anterior lumbar interbody fusion (ALIF) differs from posterior lumbar fusion approaches in that it largely preserves the posterior elements. Performed through a retroperitoneal or transperitoneal corridor anterior to the vertebral body, ALIF avoids direct dissection of the paravertebral muscles and generally preserves the posterior ligamentous complex and facet joints. Preservation of these structures maintains their potential contribution to posterior rotational and shear restraint, complementing the anterior support provided by the interbody cage. However, posterior element preservation alone should not be interpreted as evidence of lower adjacent-segment loading or a reduced risk of ASD. A subsidence rate of approximately 10.2% has been reported in a prospective series of standalone ALIF (33), although subsidence is influenced by multiple factors beyond posterior element preservation.

Clinically, ALIF is commonly used when anterior disc-space access and restoration of disc height or segmental alignment are desired, particularly at L5–S1. However, disruption of the anterior longitudinal ligament and annulus fibrosus also alters segmental mechanics, and standalone ALIF may provide less resistance to extension and axial rotation than the intact segment. In patients with preoperative facet instability or residual rotational instability, supplementary posterior fixation may therefore be considered. Facet arthrodesis represents one possible option for providing additional rotational restraint while preserving much of the posterior ligamentous anatomy.

Oblique lumbar interbody fusion (OLIF), like ALIF, generally preserves the posterior ligamentous and facet structures. OLIF is generally performed through a retroperitoneal antepsoas or prepsoas corridor anterior to the psoas muscle, rather than through the muscle itself, and therefore avoids direct surgical disruption of the posterior ligamentous complex and facet joints. The approach permits placement of a relatively large interbody cage for indirect decompression, restoration of disc height, and correction of coronal or sagittal alignment (34). Although OLIF preserves the posterior elements more directly than TLIF, this anatomical difference does not necessarily translate into lower adjacent-segment loading. In the finite element study by Wang et al., both TLIF and OLIF increased adjacent-level ROM and intradiscal pressure relative to the intact model, with no significant differences between the two fusion models (35).

The oblique cage trajectory may nevertheless influence endplate loading. In the finite element model reported by Ouyang et al., the OLIF cage configuration was associated with a 20%–30% reduction in effective endplate–cage contact area compared with the ALIF configuration, and peak endplate stress under rotational loading was higher in the OLIF model (36). These findings indicate that posterior element preservation does not eliminate other approach-specific mechanical considerations. In selected patients with residual instability or a need for additional posterior support, supplementary fixation may therefore be considered; whether facet arthrodesis improves clinical outcomes in this setting remains uncertain.

### 3.5 Posterior element disruption in TLIF and PLIF

Posterior lumbar interbody fusion (PLIF) and transforaminal lumbar interbody fusion (TLIF) provide direct posterior access for neural decompression and interbody fusion but may involve varying degrees of disruption of the posterior elements. Conventional PLIF commonly involves bilateral posterior exposure and may include laminectomy, resection of the supraspinous and interspinous ligaments, ligamentum flavum removal, paravertebral muscle dissection, and partial or bilateral facet resection, depending on the extent of decompression required. By contrast, TLIF typically uses a unilateral transforaminal corridor and may preserve more of the contralateral lamina, facet joint, and ligamentous structures, although the extent of facetectomy and ligamentous resection varies with the surgical technique and decompression requirements. Biomechanical models indicate that unilateral or bilateral facetectomy can increase segmental motion, particularly in axial rotation, with the magnitude of change depending on the extent of facet resection (12).

When posterior ligamentous and facet structures are disrupted, load sharing shifts toward the interbody cage and posterior instrumentation, which may alter bending loads and construct stiffness. In the finite element analysis by Li et al., the unilateral TLIF model showed higher peak adjacent-segment disc stress than the OLIF model with preserved posterior elements, including increases of 141.23% in extension and 71.39% in left lateral bending; stress was concentrated around the facet capsule and articular cartilage of the suprajacent segment (37). Similarly, in the finite element models reported by Cheng et al., the area of stress concentration within the adjacent disc was more than 40% greater in multilevel than in single-level PLIF constructs (38). Greater cyclic loading of the posterior instrumentation may contribute to screw–bone interface stress and implant fatigue over time. Finite element modeling has also shown greater adjacent-level stress with rigid pedicle screw constructs, consistent with redistribution of load and motion at the transition between the instrumented and adjacent segments (39). In the same finite element study, supplemental facet fixation with the FFX device was associated with a 13.5% reduction in adjacent-level facet joint stress relative to conventional pedicle screw fixation after laminectomy (39). Another finite element study reported that removal of the posterior ligamentous complex increased adjacent annular stress by more than 30% and intradiscal pressure by over 40% during flexion; these changes were reduced when posterior structural support was restored in the model (15).

PLIF and TLIF are commonly used when direct posterior neural decompression and interbody fusion are required, including selected cases of spinal stenosis, disc herniation, or nerve-root compression. The extent of posterior element disruption should therefore be considered when planning supplementary stabilization. Facet arthrodesis has been investigated as a supplementary option for providing posterior support after posterior element disruption. Prospective multicenter data on the FFX device have reported a one-year facet fusion rate of 86.3%, although these data were derived from a relatively small clinical cohort and did not establish superiority over standard pedicle screw fixation (40). Taken together, the available evidence provides a biomechanical rationale for considering facet arthrodesis as a supplementary option in selected patients with substantial posterior element disruption or residual rotational instability. However, current evidence is insufficient to recommend routine facet arthrodesis after TLIF or PLIF or to conclude that it reduces long-term symptomatic ASD.

### 3.6 Multilevel fusion and construct loading

Biomechanical changes associated with lumbar fusion may become more pronounced as the number of fused levels increases. In the finite element models reported by Cheng et al., peak pedicle screw stress in single-level PLIF was 55.0% lower than in two-level constructs and 79.4% lower than in multilevel constructs extending to the sacrum (38). Finite element analysis of OLIF by Du et al. showed a similar trend, with adjacent-segment intradiscal pressure and endplate stress in multilevel constructs increasing by 36.12% and 51.29%, respectively, compared with single-level constructs (41). Together, these models suggest that increasing fusion length may increase both adjacent-segment loading and mechanical demands on the instrumentation, particularly near the ends of the construct.

These observations provide a biomechanical rationale for considering the need for additional stabilization in selected long-segment constructs. However, current evidence is insufficient to determine whether adjunctive facet arthrodesis at the caudal end of a multilevel construct improves construct durability or reduces adjacent-segment degeneration (Table 1).

**Table 1:** Posterior element involvement and selected mechanical characteristics of common lumbar interbody fusion approaches.

Surgical approach	Posterior column disruption	Facet joint status	Biomechanical characteristics	Key risks and considerations
ALIF	Generally preserved	Preserved	Preserves posterior ligamentous and facet restraint	Standalone constructs may have limited rotational stability; subsidence and vascular risks remain (33)
OLIF	Generally preserved	Preserved	Both OLIF and TLIF increased adjacent ROM and IDP in one finite element model, without significant between-model differences (35)	Cage trajectory and endplate loading may influence construct mechanics (36)
TLIF	Variable; commonly unilateral disruption	Usually unilateral facet involvement	Facetectomy may increase rotational motion; adjacent-level stress varies with the extent of posterior disruption (12, 37)	Effects depend on decompression, facetectomy, ligamentous resection, and supplemental fixation
PLIF	Variable; commonly more extensive bilateral disruption	Partial or bilateral facet involvement	Greater posterior disruption may increase reliance on instrumentation; multilevel models show greater adjacent-disc stress concentration (38)	Mechanical effects depend on decompression extent and construct length

These patterns are descriptive rather than fixed categories; the actual extent of posterior element disruption varies with surgical technique and decompression.

### 3.7 Facet joint fusion techniques and fixation characteristics

#### 3.7.1 Dedicated facet implants and trans-facet screws

Several implant systems have been developed for facet arthrodesis, including intra-articular facet spacers and dedicated facet fusion devices. Goel proposed facet distraction using intra-articular spacers as a means of increasing foraminal dimensions and facilitating indirect neural decompression (42). In the preliminary clinical and morphometric studies cited here, facet spacer placement was associated with an increase in foraminal height of approximately 2.14 mm and an increase in interspinous distance of 5.56 mm (43, 44). The reported series also described high rates of fusion and limited segmental displacement after bilateral facet spacer fixation (43). However, these findings were derived from small early studies and should be interpreted accordingly.

The FFX device is a commercially available implant designed specifically for facet fusion. Its D-shaped, serrated titanium alloy profile is designed to provide intra-articular fixation and a surface for osseous fusion. In a prospective multicenter study of 53 patients receiving 205 implants, facet fusion was observed at 86.3% of device placements at 12 months (40). Mean visual analog scale (VAS) leg-pain scores decreased from 5.57 to 2.09, and the Oswestry Disability Index (ODI) decreased from 44.7% to 24.0%. No intraoperative or postoperative surgical complications requiring revision were reported; however, one asymptomatic device migration and eight misplaced implants were identified (40). In a separate finite element study, the FFX construct permitted approximately 0.9° of segmental motion before osseous fusion and showed lower modeled stress shielding than rigid pedicle screw constructs (39).

Facet-based screw fixation has also been evaluated in cadaveric models. Godzik et al. reported an axial rotational range of motion (ROM) of 0.88° with bilateral facet fixation, compared with 1.28 with unilateral pedicle screw fixation (45). More recently, Chin et al. compared bilateral FacetFuse trans-facet pedicle screws (TFPS) with bilateral and unilateral pedicle screw–rod constructs in fourteen cadaveric specimens. Bilateral TFPS showed ROM comparable to bilateral pedicle screw–rod fixation in extension and axial rotation and lower ROM than unilateral pedicle screw fixation across the tested loading modes (*p* < 0.001); the reported pull-out strength was approximately 694 N (46). These findings support the biomechanical feasibility of facet-based screw fixation, although cadaveric performance alone does not establish equivalence to bilateral pedicle screw fixation in clinical practice.

#### 3.7.2 Comparison between pedicle fixation and facet joint fixation

Pedicle screw fixation and facet-based fixation provide different mechanical profiles and should not be regarded as directly interchangeable. Pedicle screw–rod constructs provide high multidirectional stiffness and strong control of lateral bending; in the biomechanical study cited here, lateral-bending ROM was approximately 0.4° (45). Such rigidity is advantageous when substantial primary stability is required. By contrast, facet-based fixation acts closer to the posterior motion segment and provides substantial rotational restraint, although its control of lateral bending may be less pronounced. For example, unilateral facet fixation showed a lateral-bending ROM of approximately 3.98° in cadaveric testing (45).

Construct stiffness must therefore be interpreted in relation to the mechanical requirements of fusion rather than as an isolated measure of superiority. Adequate primary stability is necessary to limit excessive micromotion and support osseous fusion, whereas greater construct rigidity may also increase load transfer to adjacent segments. In finite element modeling, adjacent-level facet stress was approximately 13.5% higher with pedicle screw instrumentation than in the intact model, whereas stress values after facet-based fixation remained closer to those of the intact spine (39). However, a stress or stiffness profile closer to the intact condition should not be interpreted as evidence that facet fixation is clinically superior.

When combined with interbody fusion, facet fixation can supplement anterior column support by adding posterior rotational restraint, thereby forming a two-column construct. This configuration may be relevant in selected patients in whom additional posterior support is desirable without the degree of rigidity provided by bilateral pedicle screw–rod fixation. Conversely, facet fixation should not be considered a substitute for pedicle screw fixation when high primary stability is required, such as in marked instability or major deformity. The choice of fixation should therefore reflect the stability requirements of the index level, the integrity of the posterior elements, and the overall construct rather than the assumption that either greater or lower stiffness is inherently preferable (Figure 3) (Table 2).

**Figure 3:**
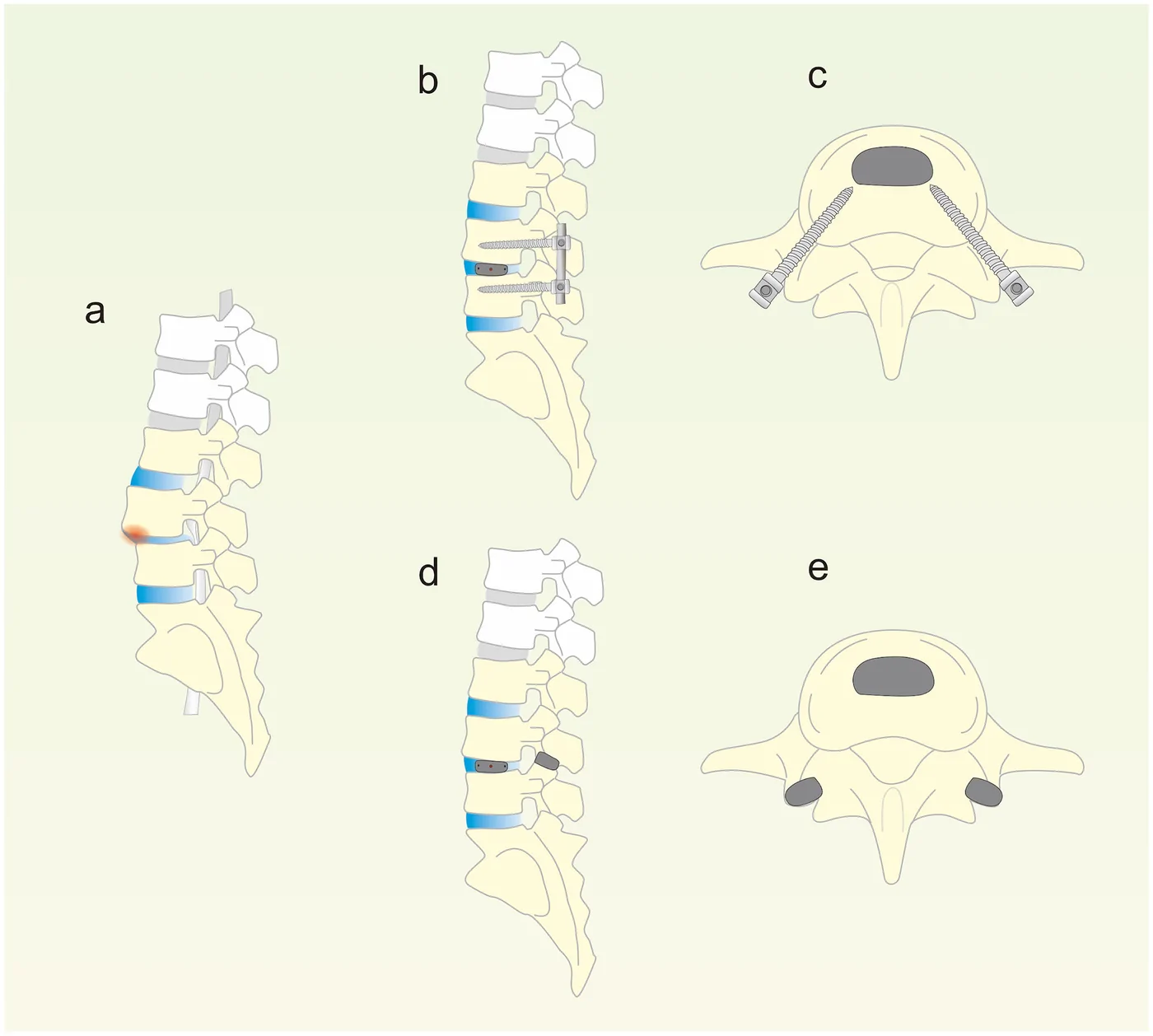
Schematic comparison of pedicle screw-rod and facet-based fixation after lumbar interbody fusion. **(a)** Preoperative sagittal view. Sagittal **(b)** and axial **(c)** views of pedicle screw-rod fixation. Sagittal **(d)** and axial **(e)** views of facet fixation.

**Table 2:** Selected biomechanical and operative characteristics of pedicle screw and facet-based fixation.

Comparison Domain	Pedicle Screw Fixation	Facet Joint Fixation
Primary Mechanical Profile	High multidirectional stiffness; strong lateral-bending control (ROM ∼0.4°) (45)	Strong rotational restraint; bilateral TFPS showed comparable extension and rotational ROM to bilateral pedicle fixation in cadaveric testing (46)
Adjacent-level Mechanics	Facet stress increased by ∼13.5% vs. the intact model in finite element analysis (39)	Adjacent-level stress remained closer to the intact model in the same finite element analysis (39)
Mechanical Limitation	Greater rigidity may increase adjacent-level load transfer (39)	Less control of lateral bending in some configurations (unilateral ROM ∼3.98°) (45)
Operative Findings	Operative time ∼152.5 min; blood loss ∼446.5 mL in one retrospective cohort (47)	Operative time ∼99.4 min; blood loss ∼251.0 mL in the same cohort (47)

Comparisons are study-specific and should not be interpreted as evidence of overall clinical superiority.

### 3.8 Evidence for combined interbody and facet fusion

Combining interbody fusion with facet fixation provides anterior interbody support together with posterior rotational restraint (Figure 4). Available studies have examined whether this configuration influences construct stability, adjacent-level loading, fusion, and perioperative outcomes, although the strength of evidence differs substantially across these endpoints.

**Figure 4:**
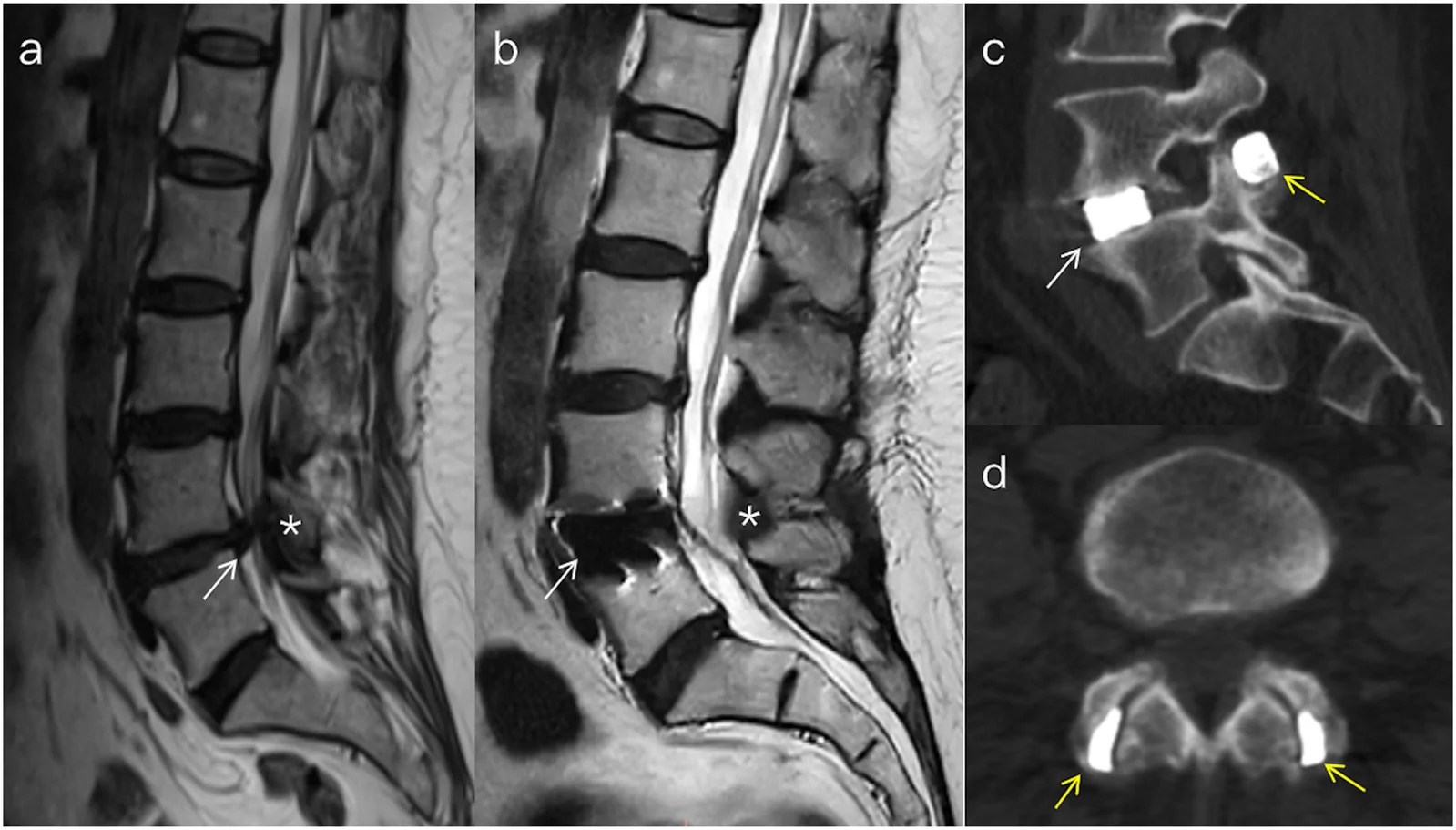
Illustrative case of L4–L5 OLIF with bilateral facet fixation. **(a)** Preoperative T2-weighted MRI. **(b)** Six-month postoperative MRI. **(c)** Sagittal CT showing the interbody cage and facet implants. **(d)** Axial CT showing bilateral intra-articular facet implant placement.

#### 3.8.1 Biomechanical effects

Biomechanical studies generally support a complementary mechanical role for facet fixation when used with interbody fusion. Cadaveric studies have shown reduced segmental motion with facet-based fixation (43, 45, 46), while finite element models suggest that facet supplementation can alter load distribution at the treated and adjacent levels (17, 39). In the finite element model reported by Sun et al., bilateral facet joint fusion fixation combined with OLIF was associated with lower adjacent-segment stress than bilateral pedicle screw fixation under several tested loading conditions, although the magnitude of the difference varied by motion (17). Simon et al. similarly reported lower adjacent-level facet stress with facet-based fixation than with conventional pedicle screw constructs (39). These findings support the mechanical feasibility of facet-based supplementation but do not establish a reduction in clinical ASD.

#### 3.8.2 Clinical and perioperative evidence

Clinical evidence remains more limited. In a prospective multicenter study of the FFX device, the one-year fusion rate was 86.3% in 53 patients, accompanied by improvements in VAS leg pain and ODI (40). However, the study was not designed to establish superiority over conventional pedicle screw fixation. In a retrospective comparative cohort, Srour reported shorter operative time and lower intraoperative blood loss with FFX fixation than with pedicle screw fixation (47). More recently, Houari et al. reported a lower long-term reoperation rate with FFX facet cages than with pedicle screw fixation, although the composite primary fusion endpoint did not differ significantly between groups (48). These findings are encouraging but remain insufficient to establish a consistent clinical advantage for facet-based supplementation across lumbar fusion procedures.

#### 3.8.3 Clinical interpretation

Facet arthrodesis may be considered as a supplementary fixation option when additional posterior rotational support is desirable, particularly when the facet joints remain available for fixation. Its role should be individualized according to the degree of posterior element disruption, residual instability, bone quality, deformity, and the stability requirements of the overall construct. Current evidence does not support routine facet arthrodesis after lumbar interbody fusion or demonstrate that it prevents symptomatic ASD.

### 3.9 Limitations of the current evidence

Finite element models and cadaveric studies provide useful mechanistic insights, but their findings cannot be directly extrapolated to clinical outcomes. One important limitation is the simplified representation of active musculature in many finite element models (49). Paraspinal muscles, particularly the multifidus and erector spinae, contribute to spinal load sharing, whereas modeling them as passive springs or omitting them may alter predicted facet and adjacent-level loads (49). Musculoskeletal models incorporating active muscle forces have reported substantial changes in adjacent-level shear loading after fusion (50).

A second limitation is the simplified representation of spinal ligaments. Many models treat ligaments as linear springs and therefore do not fully reproduce their nonlinear behavior at higher strains (51). More detailed ligament and disc formulations have shown improved agreement with experimental range-of-motion and intradiscal-pressure data, with reported correlation coefficients of 0.95–0.98 (52). Model predictions under extreme or multiplanar loading should therefore be interpreted with particular caution.

Clinical evidence for facet arthrodesis remains limited and heterogeneous. Available studies include small prospective series, retrospective comparative cohorts, and studies of different facet fixation techniques and implant designs (40, 43, 47, 48). Goel spacers, dedicated facet cages, and trans-facet screws differ in their mechanism of fixation and should not be treated as a single intervention. Differences in patient selection, concomitant interbody procedures, definitions of fusion, and follow-up duration further limit direct comparison across studies. Although recent comparative data are encouraging (48), prospective trials specifically evaluating adjunctive facet arthrodesis are still needed. Randomized trials of posterior facet arthroplasty (53, 54) provide evidence for a distinct motion-preserving strategy and should not be interpreted as direct evidence for facet arthrodesis.

The literature is also susceptible to publication bias and potential confounding from single-center or industry-associated studies. In addition, the framework used in this review to organize procedures according to the typical extent of posterior element preservation or disruption is conceptual rather than a validated classification. The actual extent of facetectomy, capsular injury, ligamentous disruption, and unilateral or bilateral posterior injury varies considerably among procedures and patients. Accordingly, this framework should not be interpreted as a prescriptive treatment algorithm.

Overall, the proposed role of facet arthrodesis should be regarded as biomechanically plausible but not yet clinically established. Current evidence does not support its routine use after lumbar interbody fusion or demonstrate that it reduces symptomatic ASD or reoperation rates. Further prospective comparative studies with standardized definitions of fusion, clinical outcomes, and posterior element injury are needed.

### 3.10 Complications and clinical considerations in lumbar facet arthrodesis

Clinical data on complications specific to lumbar facet arthrodesis remain limited. Reported concerns include surgical and implant-related complications, non-union, and adjacent segment degeneration, although much of the available evidence is derived from broader lumbar fusion literature rather than facet arthrodesis alone.

#### 3.10.1 Surgical and implant-related complications

Complications associated with facet arthrodesis depend on the surgical approach and fixation technique. General posterior lumbar complications, including dural tear, neural injury, and infection, remain relevant when facet fixation is performed together with decompression or posterior exposure, although their incidence specifically attributable to facet arthrodesis is not well established.

Implant-related complications are more technique specific. In a prospective multicenter study of 53 patients receiving 205 FFX implants, no intraoperative or postoperative surgical complications and no revision procedures were reported during the first year; however, one asymptomatic device migration (0.5%) and eight misplaced devices (3.9%) were identified (40). These findings indicate that technically successful implantation cannot be assumed even when major clinical complications are uncommon, and accurate intra-articular device placement remains an important procedural consideration.

For pedicle screw instrumentation, violation of the cranial adjacent facet joint represents a distinct concern. In a retrospective study of 148 patients undergoing single-level minimally invasive TLIF, facet joint violation was associated with greater degeneration of the superior adjacent facet and disc and with worse low-back pain and functional outcomes at follow-up, with more pronounced changes after bilateral violation (55). These observations support preservation of the cranial adjacent facet during posterior instrumentation but should not be interpreted as evidence that facet-based fixation itself prevents ASD.

#### 3.10.2 Non-union

Non-union remains an important concern after any fusion procedure. In facet arthrodesis, successful fusion depends on adequate preparation of the articular surface, graft environment, and sufficient mechanical stability. Reported fusion rates vary substantially across implant types, concomitant interbody procedures, imaging criteria, and follow-up intervals (40, 43, 48). This heterogeneity limits direct comparison between studies and reinforces the need for standardized radiographic definitions of facet fusion.

#### 3.10.3 Adjacent segment degeneration

Adjacent segment degeneration after lumbar fusion is multifactorial. Fusion-related changes in motion and load sharing may contribute to adjacent-level degeneration, but these effects interact with pre-existing degeneration, sagittal alignment, construct length, patient factors, and surgical injury to posterior structures (5–7, 56). Biomechanical changes such as increased adjacent-level motion, intradiscal pressure, and facet loading should therefore be regarded as potential mechanistic contributors rather than direct predictors of symptomatic ASD.

Reported risk factors for adjacent segment degeneration include longer fusion constructs, pre-existing adjacent-level degeneration, sagittal malalignment, patient age, and surgical factors affecting the posterior elements (5–7, 15, 16, 56). Posterior ligamentous or facet disruption may contribute to altered adjacent-level mechanics, but its independent contribution to symptomatic ASD has not been established (15, 16).

Prevention of ASD should therefore focus on appropriate patient selection, preservation of sagittal alignment, avoidance of unnecessary fusion extension, and preservation of posterior structures when compatible with the required decompression and surgical goals. When substantial posterior element disruption is unavoidable, the need for supplementary fixation should be assessed according to residual stability and overall construct requirements. Whether facet arthrodesis specifically reduces symptomatic ASD remains unknown.

## 4 Discussion

This review considers posterior element preservation and disruption as one factor that may influence the biomechanical environment after lumbar interbody fusion. Previous work has highlighted the potential contribution of iatrogenic ligamentous, muscular, and facet injury to adjacent-segment degeneration (15, 16, 57). Building on this literature, we organize the available evidence according to the typical pattern and extent of posterior element involvement associated with different surgical procedures. This approach is intended as a descriptive framework for interpreting biomechanical evidence rather than a validated classification or prescriptive treatment algorithm.

The clinical implications of this framework are necessarily conditional. ALIF and OLIF generally preserve the posterior ligamentous and facet structures, whereas TLIF and PLIF may involve greater posterior disruption depending on the extent of decompression, facetectomy, and ligamentous resection. In procedures with preserved posterior elements, additional fixation may be unnecessary when adequate stability is achieved with the interbody construct alone. When substantial posterior structures have been removed or residual rotational instability remains, supplementary posterior fixation may be considered. Facet arthrodesis represents one possible option in such selected cases, but current evidence does not support its routine use after TLIF or PLIF. In multilevel constructs, where finite element studies suggest increasing mechanical demands on adjacent segments and instrumentation (38, 41), the need for supplemental fixation should likewise be determined by the stability requirements of the overall construct rather than by fusion length alone.

L5–S1 warrants particular consideration because of its distinct lumbosacral anatomy and mechanical environment. Facet orientation becomes progressively more coronal toward the lower lumbar spine, with relatively coronal orientation reported at the lumbosacral junction (25, 26). Because facet orientation influences rotational and shear restraint, posterior element integrity may be particularly relevant when fusion is performed at this level (11, 22). Anterior approaches such as ALIF can preserve the posterior facet and ligamentous structures, whereas posterior decompression or facetectomy may alter their contribution depending on the extent of resection. Finite element modeling has also suggested that facet-based fixation combined with OLIF may reduce L5–S1 disc stress relative to bilateral pedicle screw fixation under selected loading conditions (17). However, this remains a surrogate mechanical finding and does not establish improved fusion, patient-reported outcomes, or lower rates of symptomatic ASD specifically at L5–S1. Decisions regarding supplemental fixation at the lumbosacral junction should therefore be based on residual stability, posterior element integrity, construct length, and the overall mechanical requirements of the fusion rather than on the level alone.

The comparison between facet-based and pedicle screw fixation should also avoid treating construct stiffness as a simple measure of superiority. Pedicle screw constructs provide greater multidirectional rigidity and remain appropriate when high primary stability is required, whereas facet-based fixation may provide substantial rotational restraint with a different stiffness profile (39, 45, 46). Adequate stability is necessary to limit excessive micromotion and support osseous fusion, while unnecessary rigidity may increase load transfer to adjacent segments. Thus, a construct that more closely approximates intact-spine stiffness is not inherently superior; the relevant goal is an appropriate balance between index-level stability and stress transfer to adjacent motion segments.

Clinical evidence for facet arthrodesis remains preliminary. Prospective multicenter data have reported favorable fusion and patient-reported outcomes with the FFX device (40), while retrospective comparative studies suggest potential reductions in operative time and blood loss relative to pedicle screw fixation (47). More recent long-term comparative data reported a lower reoperation rate with FFX facet cages, although the primary fusion endpoint did not differ significantly between groups (48). In parallel, finite element studies have reported lower adjacent-level stress with facet-based fixation under selected loading conditions (17, 39). These findings support continued clinical evaluation but do not establish that facet arthrodesis improves fusion rates, patient-reported outcomes, or symptomatic ASD compared with conventional fixation.

The proposed framework is most applicable to index surgery and should not be directly extrapolated to revision procedures. In revision surgery, the original pattern of posterior-element preservation or disruption may no longer reflect the current mechanical environment. Previous decompression or facetectomy may partially remove or substantially alter the facet joints, whereas prior fusion or instrumentation may change load sharing and the stability requirements of the revised construct. Consequently, the concept of combining anterior interbody support with facet-based posterior stabilization is relevant only when the facet joints remain anatomically available for fixation and when the mechanical requirements of the revision construct can be adequately addressed by this strategy. When the posterior anatomy has been substantially altered or greater multidirectional stability is required, the choice of supplemental fixation should instead be determined by the remaining posterior structures, fusion status, existing implants, and the overall mechanical demands of the reconstruction. Revision surgery should therefore not be approached according to the original surgical procedure alone; rather, the current anatomy and residual stability should guide reconstruction planning. Evidence specifically evaluating facet arthrodesis in revision lumbar surgery remains limited, and its role in this setting should be regarded as individualized rather than routine.

Future research should prioritize prospective comparative studies of interbody fusion with and without adjunctive facet arthrodesis, using standardized definitions of facet fusion and clinically meaningful outcomes such as patient-reported outcomes, symptomatic ASD, and reoperation. Biomechanical studies should incorporate active muscle forces, patient-specific anatomy, and nonlinear ligament behavior to improve model validity (49–52). Further work is also needed to determine whether specific patterns of posterior element disruption, segmental anatomy, or preoperative instability can identify patients most likely to benefit from facet-based supplementation.

## 5 Conclusion

The facet joints and posterior ligamentous structures contribute importantly to lumbar segmental stability and load sharing. Their disruption during lumbar fusion may alter postoperative biomechanics, including segmental motion, construct loading, and stress transfer to adjacent levels. However, adjacent segment disease is multifactorial, and the independent clinical contribution of posterior element injury remains uncertain.

Facet-based fixation provides rotational restraint and may complement interbody support in selected patients, particularly when substantial posterior element disruption or residual instability creates a need for additional posterior stabilization. Its mechanical role differs from that of pedicle screw fixation, which remains appropriate when greater multidirectional stability is required. A stiffness profile closer to that of the intact spine should therefore not be interpreted as evidence of clinical superiority.

Current evidence for adjunctive facet arthrodesis is derived largely from finite element studies, cadaveric experiments, and relatively small clinical series. Although these studies support biomechanical feasibility and report encouraging fusion and perioperative outcomes, they do not establish that routine facet arthrodesis after TLIF or PLIF improves clinical outcomes or reduces symptomatic ASD or reoperation. The condition of the posterior elements may therefore be considered as one component of surgical decision-making, but prospective comparative studies are needed before specific treatment recommendations can be established.
